# Free Food and Laziness

**Published:** 1907-09-21

**Authors:** W. Carlile

**Affiliations:** Honorary Chief Secretary. The Church Army Headquarters, 55 Bryanston Street


### EDITOR'S LETTER-BOX.

**[Our Correspondents are reminded that prolixity is a great bar to publication, and that brevity of style and conciseness of statement greatly facilitate early insertion.]**

### FREE FOOD AND LAZINESS.

Sir,—In commenting upon the last annual census of London's homeless poor in your issue of August 31 you remark that a considerable number of the homeless were receiving food "without any inquiry as to the cause of the hunger." While the King's Labour Tents were in operation under our direction last winter 40,000 homeless men received food, and in most cases a bed in return for work. In every case they were hungry—desperately so, as a rule—and in every case the cause of the hunger was unemployment. Surely it is better to give these men the opportunity of working (the very acceptance of which is some guarantee of their genuine inability to find work elsewhere) than to thrust them as a last resource upon Poor Relief with its ineradicable taint of pauperism.

In our permanent Labour Homes and other institutions we do indeed hope to achieve spiritual as well as physical regeneration, for it is only by building up the Whole Man that we can hope to return him to the world a self-supporting and useful member of society. So many of our "cases" keep in touch with us long after leaving us to take up the battle of life for themselves anew that we are able to say with assurance about half do in the future lead honest industrious lives. And we help 400,000 annually!

As to the risk of breeding a race of loafers, which you seem to fear, we help only those who help themselves, and adhere rigidly to the Bible maxim that "if any man would not work, neither should he eat." I am, sir,

Your obedient servant,
W. Carlile,
Honorary Chief Secretary.

The Church Army Headquarters,
55 Bryanston Street, September 5, 1907.

[It is gratifying to learn from Prebendary Carlile's letter that the Church Army is not among the agencies which give food without work. We fear that all charitable bodies are not so wise. To state, however, that "in every case the cause of the hunger was unemployment" as a solution of the point we raised is hardly fair. We do not imagine that a sane man in regular well-paid employment would go to the Church Army or any other agency to beg for a meal. The real point is, "What was the cause of the unemployment?" There are so many possible reasons—change in the conditions of an industry, old age, or ill-health among good reasons, and vice, laziness, or incompetence among bad ones. And every one of these requires different treatment. Doubtless the officials of the Church Army appreciate the difficulty of the problem, and their success in keeping in touch with those they have helped shows that they are able to deal with it. But those who, like the writer, have Poor-law experience, know that readiness to undertake test work is not always a proof of genuine distress. The test must be fitted to the weak, and the strong can often do it easily, and thereby gain more than the same effort would secure in the ordinary labour market. This fact is a cause of endless difficulty in dealing with the unemployed. Many a loafer prefers a few hours in a stoneyard or some similar place to the strain of regular work done day in, day out. And always there are those who undertake work under Poor-law or charitable agencies when, and only when, begging has been unsuccessful. As to the "ineradicable taint of pauperism" contained in Poor-law relief, our experience is that those seem to feel it most whose record can least well stand the scrutiny of Poor-law officials. It is not less degrading to take help from voluntary donations than from that organisation to which every citizen, by reason of his citizenship, has a right. In London, at least, those who administer the Poor-law are in no way inclined to be too hard on the respectable but unfortunate poor; and it is eminently desirable, in the interests of society, that there should be genuine understanding, sympathy, and correlation between the Voluntary and the Poor-law agencies, the object of both of which is the alleviation of distress and the raising of the recipients to the position of self-supporting citizens.—Ed. The Hospital.]

THE HOSPITAL *September* 21, 1907.

